# Cerebrospinal Fluid sCD27 as a Biomarker of Neuroinflammatory Disease: A Systematic Review and Meta‐Analysis

**DOI:** 10.1111/jnc.70451

**Published:** 2026-04-28

**Authors:** Nadia Damholt Savino, Malene Bredahl Hansen, Amanda Marie Lund Christiansen, Sahla El Mahdaoui, Finn Sellebjerg, Jeppe Romme Christensen

**Affiliations:** ^1^ Danish Multiple Sclerosis Center Copenhagen University Hospital—Rigshospitalet Glostrup Denmark; ^2^ Department of Health and Medical Sciences University of Copenhagen Copenhagen Denmark

## Abstract

Neuroinflammatory diseases of the central nervous system (CNS) present considerable diagnostic challenges due to overlapping clinical features and the lack of specific biomarkers capable of reliably detecting CNS inflammation. Soluble CD27 (sCD27) is a marker of adaptive immune activation, released upon CD27–CD70 interaction. sCD27 has emerged as a promising cerebrospinal fluid (CSF) biomarker, but its clinical utility remains unclear. This systematic review and meta‐analysis aimed to clarify the diagnostic value of CSF sCD27 across neuroinflammatory conditions. We systematically searched PubMed, Embase, and Scopus for studies reporting CSF sCD27 levels in neuroinflammatory disorders versus controls, including demyelinating diseases, autoimmune encephalitis, neuroinfectious diseases, and primary CNS lymphoma, following PRISMA 2020 guidelines. Nineteen studies met the inclusion criteria for qualitative synthesis, and ten provided sufficient quantitative data for meta‐analysis, encompassing 685 neuroinflammatory and 751 control participants. Using multivariate and random‐effects models, we found significantly elevated levels of CSF sCD27 in neuroinflammatory diseases compared to controls (standardized mean difference [SMD] = 1.24, 95% CI 0.98–1.51, *p* < 0.0001), with consistent results in sensitivity and subgroup analysis restricted to multiple sclerosis. Despite between‐study heterogeneity, largely driven by variation in assay methods, reporting units, and study populations, effect sizes remained large and robust. Most studies also reported excellent diagnostic accuracy, with area under the curve (AUC) values above 0.85, supporting the discriminatory potential of CSF sCD27 for neuroinflammatory diseases versus controls. Collectively, these findings strongly support that CSF sCD27 is a robust biomarker of adaptive immune‐mediated neuroinflammation across a spectrum of neuroinflammatory diseases. Future research should focus on assay standardization and consistent reporting practices using well‐characterized prospective cohorts of a broader spectrum of neuroinflammatory disorders to define clinical thresholds and facilitate the integration of CSF sCD27 into diagnostic protocols. This study provides a comprehensive synthesis and substantiates CSF sCD27 as a promising biomarker for detecting adaptive immune‐mediated neuroinflammation in clinical practice.

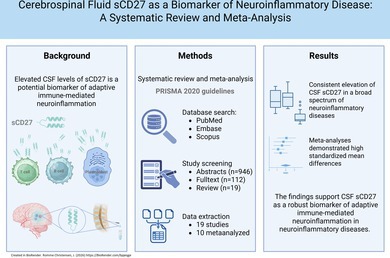

## Graphical abstract

Neuroinflammatory diseases of the CNS are difficult to diagnose, and current CSF biomarkers lack sensitivity across conditions. Soluble CD27 (sCD27), a marker of adaptive immune activation, has emerged as a promising candidate, but its clinical utility remains uncertain. In this systematic review and meta‐analysis, we evaluated CSF sCD27 across 19 studies and demonstrated consistently elevated levels in a spectrum of neuroinflammatory diseases. The findings strongly support that CSF sCD27 robustly distinguishes inflammatory from noninflammatory conditions, supporting its relevance as a generic biomarker of CNS inflammation and its potential integration into diagnostic workflows.

**Abbreviations**
AUC: area under the curve
CD27: cluster of differentiation 27
CIS: clinically isolated syndrome
CNS: central nervous system
CSF: cerebrospinal fluid
CXCL‐13: C‐X‐C motif chemokine ligand 13
ECLIA: electrochemiluminescence immunoassay
ELISA: enzyme‐linked immunosorbent assay
HC: healthy controls
IgG: immunoglobulin G
IND: inflammatory neurological disease
IQR: interquartile range
kDa: kilodalton
MS: multiple sclerosis
NHLBI: National Heart, Lung, and Blood Institute
NIND: non‐inflammatory neurological disease
NMOSD: neuromyelitis optica spectrum disorder
NR: not reported
OCB: oligoclonal band
PCNSL: primary central nervous system lymphoma
PRISMA: Preferred Reporting Items for Systematic Reviews and Meta‐Analyses
RRMS: relapsing‐–remitting multiple sclerosis
SAS: spinal anesthesia subjects
SC: symptomatic controls
sCD27: soluble CD27
SD: standard deviation
SMD: standardized mean difference
SPMS: secondary progressive multiple sclerosis

## 1 Introduction

Neuroinflammatory diseases form a broad group of conditions characterized by inflammation within the central nervous system (CNS). This group includes demyelinating disorders, various infectious and parainfectious CNS disorders, autoimmune encephalitis, neurosarcoidosis, CNS manifestation of hematological diseases, and many others. These conditions pose considerable diagnostic challenges as their clinical presentations are often non‐specific and frequently overlap with other neurological disorders, making timely and accurate diagnosis difficult. Current routine biomarkers of neuroinflammation, such as cerebrospinal fluid (CSF) cell count, intrathecal IgG synthesis, CSF C‐X‐C motif chemokine ligand 13 (CXCL13), and contrast‐enhancing lesions, have insufficient sensitivity for detecting neuroinflammation across all neuroinflammatory diseases (Arun et al. 2020; Bromberg et al. 2007; Erhart et al. 2023; Jarius et al. 2011, 2020; Komori et al. 2015). Consequently, diagnostic delays are common and may lead to prolonged hospitalization, misdiagnosis, unnecessary diagnostic testing, or inappropriate treatment (Dubey et al. 2018; Knudtzen et al. 2017; Street et al. 2014; Zürrer et al. 2024). Given these challenges, early and accurate detection of neuroinflammation is essential to improve clinical outcomes. This underscores the need for reliable biomarkers specifically capable of identifying CNS inflammation at an early timepoint in the diagnostic process. However, existing biomarkers frequently lack the necessary sensitivity and disease specificity to effectively guide diagnosis and treatment decisions (Hegen et al. 2023).

Cluster of differentiation 27 (CD27), also known as tumor necrosis factor receptor superfamily member 7, is a transmembrane receptor and is expressed on T cells, B cells, and natural killer cells (Grimsholm 2023; Han et al. 2016; Watts et al. 2025). Interaction with its ligand, CD70, promotes T cell survival, proliferation, and differentiation. T cell activation through the T cell receptor/CD3 complex leads to upregulation of CD27 expression and subsequent cleavage of the CD27 receptor, resulting in the release of a soluble form known as soluble CD27 (sCD27) (de Jong et al. 1991; Hintzen, de Jong, et al. 1991; van Lier et al. 1987). This soluble molecule has a molecular weight of approximately 32 kDa and can be detected in both serum and CSF. Since its discovery in 1991, sCD27 has been recognized as a marker of adaptive immune activation (Cencioni et al. 2024; El Mahdaoui et al. 2023; Hintzen, de Jong, et al. 1991; Hintzen et al. 1995; Komori et al. 2015).

Elevated CSF levels of sCD27 have shown strong potential as a biomarker of adaptive immune‐mediated neuroinflammation in numerous studies, particularly in demyelinating disorders such as multiple sclerosis (MS), but also in other conditions including autoimmune encephalitis, neuroborreliosis, and CNS lymphoma (Cobanovic et al. 2024; Feresiadou et al. 2019; Komori et al. 2015; Mahler et al. 2020; Murase et al. 2000; van der Vuurst de Vries et al. 2017; Wong et al. 2018). Despite this growing body of evidence, the clinical utility of sCD27 remains uncertain, largely due to heterogeneity in study populations and assay methodologies. As a result, no standardized diagnostic thresholds or established clinical guidelines for CSF sCD27 have been defined to date.

To address these uncertainties and clarify the clinical utility of CSF sCD27 as a biomarker of adaptive immune‐mediated neuroinflammation, we conducted a systematic review and meta‐analysis. The objective was to determine whether elevated CSF sCD27 levels reliably predict neuroinflammation across the spectrum of neuroinflammatory diseases.

## 2 Methods

### 2.1 Registration and Protocol

This review was conducted in accordance with the Preferred Reporting Items for Systematic Reviews and Meta‐Analyses (PRISMA) 2020 guidelines (Page et al. 2021). A detailed study protocol outlining the review objectives, eligibility criteria, search strategy, and planned methods for data extraction and analysis was developed prior to initiating the review process. The full protocol is provided in Supporting Information.

### 2.2 Eligibility Criteria

We included original research articles published in peer‐reviewed journals with full‐text availability in English. Eligible study designs encompassed retrospective, prospective, and cross‐sectional studies. Studies were considered if they reported CSF sCD27 levels in cohorts of patients diagnosed with neuroinflammatory diseases compared to control groups without neuroinflammation, such as healthy controls (HC), symptomatic controls (SC), spinal anesthesia subjects (SAS), or individuals with non‐inflammatory neurological disorders (NIND). No age restrictions were applied.

For this review, pathological neuroinflammation was defined based on current scientific consensus as an inflammatory process characterized by the presence of activated immune cells and proinflammatory mediators (Buckley and McGavern 2022; DiSabato et al. 2016; Teleanu et al. 2022). Accordingly, neuroinflammatory diseases included: inflammatory demyelinating disorders, autoimmune encephalitis, neuroinfectious diseases, neurosarcoidosis, and CNS lymphoma. Neurodegenerative diseases were classified as non‐inflammatory, with the exception of Parkinson's and Huntington's disease, whose underlying pathophysiology remains uncertain; these were therefore excluded from both neurodegenerative and neuroinflammatory categories. In studies that included multiple disease or control groups, any groups with unclear neuroinflammatory status were excluded from the review, while the remaining groups were retained. Control groups with evident systemic inflammation, which could potentially affect CSF sCD27 levels, were also excluded.

Studies were eligible for inclusion in the systematic review if they reported at least one of the following outcomes related to CSF sCD27:

− Mean or median concentration values
− Area under the curve (AUC) values with or without sensitivity and specificity data
− Fold‐change values compared to controls.

Only studies reporting mean or median CSF sCD27 concentrations with corresponding standard deviations (SD) or interquartile ranges (IQR) were included in the subsequent meta‐analysis. In studies with multiple disease groups stratified by treatment status, only the untreated groups were included.

### 2.3 Search Strategy

A comprehensive literature search was conducted by author NS across three databases: PubMed, Embase, and Scopus. The search strategy combined controlled vocabulary (e.g., MeSH terms) and free‐text keywords related to sCD27 and neuroinflammatory diseases, with the aim of identifying studies reporting CSF sCD27 levels in relevant patient populations. Filters were applied to only include original research articles published in English with full‐text availability. No restrictions were placed on publication date. The complete PubMed search strategy is provided in the Supporting Information. All databases were last queried on February 7, 2025.

### 2.4 Study Selection

Titles and abstracts identified through the literature search were independently screened by two reviewers (NS and AC) using the Rayyan web application for systematic reviews (Ouzzani et al. 2016). Following this initial screening, the same reviewers independently assessed the full texts to determine eligibility based on predefined criteria. Any disagreements were resolved through discussion, and if consensus could not be reached, a third reviewer (JRC) was consulted.

### 2.5 Data Extraction

Data extraction was performed independently by two reviewers (NS and MBH). In cases of disagreement, a third reviewer (JRC) was consulted to reach consensus. Extracted data included: study characteristics, participant demographics, disease profiles, patient treatment status, CSF sCD27 measurement methods, CSF sCD27 levels, and diagnostic accuracy metrics. Missing data were recorded as not reported (NR). When critical data were incomplete or unclear, corresponding authors were contacted to request the necessary information.

### 2.6 Risk of Bias Assessment

The methodological quality of included studies was assessed using the National Heart, Lung, and Blood Institute (NHLBI) Quality Assessment Tool for Observational Cohort and Cross‐Sectional Studies (NHLBI 2013). Domains covering study population, exposure and outcome measurement, confounding, and statistical analysis were evaluated. For the purposes of this review, the tool was adapted to define exposure as the presence of a neuroinflammatory disease and the outcome as CSF sCD27 levels. Two reviewers (NS and MBH) independently conducted the quality assessments. Any discrepancies were resolved through discussion or, when necessary, in consultation with a third reviewer (JRC). Results were visualized using the robvis tool, employing traffic light plots to summarize the risk of bias across studies (McGuinness and Higgins 2021).

### 2.7 Data Synthesis and Statistical Analysis

All extracted data from the included studies were organized systematically to enable both descriptive and quantitative synthesis. For the studies included in the meta‐analysis, values reported as medians with IQRs or ranges were converted to means and SDs using the methods described by Luo et al. (2018) and Wan et al. (2014). Standard errors were converted to SDs when applicable. Demographic and CSF sCD27 data were grouped into disease and control cohorts. Within each subgroup, weighted means and SDs were calculated based on the sample size. Pooled weighted means were subsequently derived across all disease and control groups.

For the meta‐analysis of CSF sCD27 levels, two approaches were used. First, a multivariate random‐effects model was applied, incorporating a variance–covariance matrix to account for shared control or disease groups across subgroups. Second, subgroup data within each study were collapsed to create a single disease and control group per study, followed by a conventional random‐effects model. In both analyses, effect sizes were calculated as standardized mean differences (SMD) with 95% confidence intervals (CI). A random‐effects model was chosen due to clinical and methodological heterogeneity across studies.

Heterogeneity was assessed using Cochran's Q test and reported with corresponding *p*‐values. Sensitivity analyses were performed by excluding individual studies to assess the robustness of the pooled estimates. Forest plots were used to visualize meta‐analytic results. All statistical analyses were conducted in R version 4.4.2, using the metafor package (R Core Team 2024; Viechtbauer 2010). A two‐sided *p*‐value of < 0.05 was considered statistically significant.

In line with current recommendations, publication bias was not assessed using funnel plots or asymmetry tests due to the limited number of studies included in the meta‐analysis (*n* = 10).

### 2.8 Ethical Considerations

This synthesis only used data from published studies, and ethical approval was therefore not required.

## 3 Results

### 3.1 Study Selection

**FIGURE 1:**
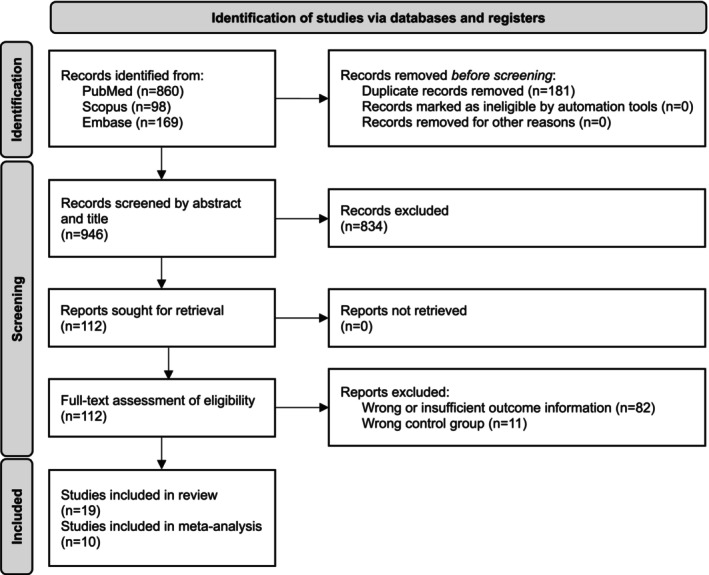
Study selection flow diagram. Preferred Reporting Items for Systematic Reviews and Meta‐Analyses (PRISMA) 2020 flow diagram showing the study selection process for the systematic review and meta‐analysis. Adapted from Page et al. (2021).

The initial search yielded 1027 records. After removing duplicates, 986 records underwent abstract screening, and 112 full‐text articles were assessed for eligibility. Nineteen studies met the inclusion criteria and were included in the systematic review. Of these, 10 studies provided sufficient data for meta‐analysis (Figure 1).

The study by El Mahdaoui et al. (2023) was excluded from the meta‐analysis due to overlapping cohorts with Mahler et al. (2020). The remaining eight studies were excluded from the meta‐analysis due to insufficient data on CSF sCD27 levels.

### 3.2 Study Characteristics

Study characteristics for all studies included in this systematic review, comprising study design, population details, demographics, immunomodulatory treatment status, CSF sCD27 measurement methods, and CSF sCD27 levels, are summarized in Table S1. An overview of population details, immunomodulatory treatment status, and CSF sCD27 levels is presented in Table 1.

**TABLE 1:** Overview of key data extracted for all included studies.

Author	Cases: *n*	Controls: *n*	Immunomodulatory treatment	CSF sCD27 level
Liu et al. (2018)	NMOSD: 31 RRMS: 23	NIND: 22	No treatment at sample time. Samples taken during a relapse	sCD27 mean: NMOSD: 20.46 RRMS: 13.24 NIND: 10.35
Murase et al. (2000)	PCNSL: 13 IND: 12	OBT: 30	PCNSL: All: % corticosteroid within 1 week 10: during treatment 4: during remission 12: treatment naïve OBT + IND: treatment naive	sCD27 mean: PCNSL (26 samples): 123.5 OBT: 4.2 IND: 74.4
Hintzen, van Lier, et al. (1991)	MS: 19 *‐RRMS:16* *‐PPMS: 3* IND: 26	NIND: 56	No treatment within 6 months prior to sampling	sCD27 median: MS: 63 NIND: 7 IND: 44
Mondria et al. (2008)	SPMS+ BMT:14 RRMS: 12	NIND: 17	SPMS: 10 samples pre BMT, 8 samples post BMT RRMS: NR NIND: NR	sCD27 median: SPMS preBMT: 112 SPMS postBMT: 55 NIND: 4.0 RRMS: 54.5
Hintzen et al. (1999)	MS: 41 *‐RRMS: 30* *‐SPMS: 11*	NIND: 43	NR	sCD27 mean: NIND: 3 MS: 46
Lundblad et al. (2023)	RRMS AHSCT+: 45	HC: 32	CSF samples are taken pre – and post AHSCT. Pre AHSCT: DMT+: 35 DMT‐: 10	sCD27 median: RRMS *‐Pre AHSCT: 352* *‐Post AHSCT (1y): 143* *‐Post AHSCT (2y): 120* HC: 63
Feresiadou et al. (2019)	IND: 338	NIND: 338 Controls: 127 *‐HC:47* *‐SC: 38* *‐SAS: 42*	NR	sCD27 median: Controls: 64 *‐HC 16* *‐SC 13* *‐SAS 252* NIND 58 IND 740
Cobanovic et al. (2024)	NMDA AE: 21 *‐untreated: 12* NMDA AE+: 5 ‐untreated: 4* LGI1 AE: 14 *‐untreated: 8* *3 herpes encephalitis, 1 CNS lymphoma	SC: 37	A subgroup of untreated patients is used in analyzation of sCD27	sCD27 median in untreated subgroups: LGI1 AE: 551 NMDA AE: 1571 NMDA+ AE: 19988 SC: 250
Mahler et al. (2020)	Treated MS: 13 *‐NZB: 6* *‐AZB: 7* Untreated MS: 22 *‐CIS: 6* *‐RRMS: 16*	SC: 34	MS patients are divided in a treated and untreated group	sCD27 median: Treated MS: 732 Untreated MS: 2460 SC: 270 sCD27 mean: Treated MS: 985 Untreated MS: 3360 SC: 285
Blok et al. (2025)	PPMS:104 RRMS: 38	AD: 22	1 RRMS +1 PPMS had corticosteroids within 4 weeks of CSF sample, otherwise treatment naive	sCD27 mean: PPMS (27 CSF samples): 2444 RRMS (29 CSF samples): 2893 ad (20 CSF samples): 213
Komori et al. (2015)	PPMS: 81 SPMS: 74 RRMS: 121 OIND: 45	HC: 8 NIND: 57	No treatment min. 3 months prior to CSF collection (exception of a few OIND, not further specified)	sCD27 median: Exact result can't be read from Figure 4, but significantly higher levels in RRMS+PPMS+SPMS+OIND vs. HC+ NIND
Kersten et al. (1996)	PCNSL: 4	SC: 50	NR (only info regarding 1 PCNSL patient: 1 sample pretreatment and 1 sample posttreatment)	sCD27 median: SC: 1.66 PCNSL: 63
El Mahdaoui et al. (2023)	RRMS: 40* *17 RRMS are used in Mahler et al.	SC: 9* *8 SC are used in Mahler et al.	Treatment naive RRMS patients	sCD27 median: RRMS: 1020 SC: 130
Kara et al. (2007)	30 cases *‐ALL: 18* *‐NHL: 7* *‐AML: 5*	NIND: 5	CSF samples obtained for diagnosis or upon therapeutic intrathecal administration of cytotoxic drugs. Treatment status not further specifies	sCD27 mean: In case group overall: *‐LI + (6): 126.44* *‐LI – (24): 68.05* NIND: no exact value, in article written below cutoff value (350)
Panackal et al. (2017)	Cryptococcal spinal arachnoiditis*: 6 *IND in this review	HC: 11	All patients were treated with antifungal therapy at sampling. Samples pre – and post immunomodulatory treatment	sCD27 median: IND: 153 HC: 8
Åkesson et al. (2023)	Discovery cohort: MS: 92 *‐RRMS: 30* *‐CIS: 62* Replication cohort: MS: 51 *‐RRMS: 30* *‐CIS: 21*	Discovery cohort: HC: 23 Replication cohort: HC: 20	DC:DMT within 3 m before baseline: 5 +, 87—Steroid treatment within 3 m before baseline: 9 +, 83—RC:DMT within 3 m before baseline: 0 +, 51—Steroid treatment within 3 m before baseline: 2 +, 49—	DC: Log2FC = 2.77 RC: Log2FC = 2.73
Hinsinger et al. (2024)	Verification cohort: SC‐CIS: 15 FC‐CIS: 15 RRMS: 30 PPMS: 14 IND: 13 ION: 15 PIND: 14	Verification cohort: SC: 30 NIND: 13	NR	Verification cohort: sCD27 FC: NIND vs. SC: 1.32 PIND vs. SC: 1.35 IND vs. SC: 3.66 RRMS vs. SC: 10.64 PPMS vs. SC: 5.16 RRMS vs. IND: 2.91 RRMS vs. NIND: 8.06
Held et al. (2025)	Discovery cohort: RRMS: 29 PPMS: 30 LNB: 8 Verification cohort: RRMS: 10 PPMS: 10	Discovery cohort: NC: 20 Verification cohort: NC: 8	MS patients were treatment naive	VC ± DC: sCD27 log2FC: MS vs. NC: 2.88
Tan et al. (2024)	Cohort 1: RRMS: 25 SPMS: 25 PPMS: 8	Cohort 1: NIND: 23	No DMT at inclusion	sCD27 NPX: NIND: 4.65 MS: 6.42 *Calculated FC MS* versus *NIND: 3,42*

*Note:* Summary of population details, immunomodulatory treatment status, and CSF sCD27 levels for all included studies.

Abbreviations: AD, Alzheimer's disease; AE, autoimmune encephalitis; AE+, autoimmune encephalitis with additional neuroinflammatory diagnoses; AHSCT, autologous hematopoietic stem cell transplantation; ALL, acute lymphoblastic leukemia; AML, acute myelogenous leukemia; BMT, bone marrow transplant; CIS, clinically isolated syndrome; CNS, central nervous system; CSF, cerebrospinal fluid; DMT, disease modifying therapy; FC, fast converting; HC, healthy controls; IND, inflammatory neurological disease; ION, isolated opticus neuritis; LGI1, leucine‐rich glioma‐inactivated 1; LI, leptomeningeal involvement; LNB, Lyme Neuroborreliosis; Log2FC, Log2 fold change; MOGAD, myelin oligodendrocyte glycoprotein–associated disease; MS, Multiple Sclerosis; n, number of participants per study group; NC, Neurologic controls; NIND, noninflammatory neurological disease; NHL, Non‐Hodgkins lymphoma; NMDA, N‐methyl‐D‐aspartate receptor; NMOSD, neuromyelitis optica spectrum disorder; NR, not reported; NPX, normalized protein expression; OBT, other brain tumors; OIND, other inflammatory neurological disease; PCNSL, primary central nervous system lymphoma; PIND, peripheral inflammatory neurological disease; PPMS, primary progressive multiple sclerosis; RRMS, relapsing–remitting multiple sclerosis; SAS, spinal anesthesia subjects; SC, slow converting; sCD27, soluble CD27.

#### 3.2.1 Design and Populations

Twelve studies employed a retrospective design, six were prospective, and one study did not clearly specify its design. The neuroinflammatory disease groups included MS, neuromyelitis optica spectrum disorder (NMOSD), autoimmune encephalitis, primary central nervous system lymphoma (PCNSL), cryptococcal spinal arachnoiditis, neuroborreliosis, and a mixed inflammatory category commonly referred to as inflammatory neurological disease (IND). The majority of studies focused on MS, often further subclassified into clinically isolated syndrome (CIS), relapsing–remitting MS (RRMS), secondary progressive MS (SPMS), and primary progressive MS (PPMS). Control groups varied and comprised HC, SAS, SC, and NIND, with these control categories being evenly represented across the studies (Teunissen et al. 2013). The NIND group comprised a wide range of non‐inflammatory diagnoses (e.g., stroke, dementias, movement disorders, radiculopathy, leukodystrophies, metabolic neurological diseases, headache disorders) which varied across studies.

#### 3.2.2 CSF sCD27 Detection Methods

Studies employed various methods to detect CSF sCD27 levels. Eleven studies used enzyme‐linked immunosorbent assays (ELISA), and three used electrochemiluminescence immunoassays (ECLIA). Of these, eleven used commercially available kits, while three applied in‐house ELISA or ECLIA protocols. One study did not clearly report the method of detection (Panackal et al. 2017). The use of different ELISA assays led to variation in measurement units: eight studies reported CSF sCD27 in units per milliliter (U/mL), while seven used picograms per milliliter (pg/mL). Among the studies included in the meta‐analysis, five studies used U/mL and five used pg/mL. The remaining four studies utilized proteomics‐based methods for protein quantification; three of these employed the Olink platform. Only one of the four proteomics studies additionally quantified CSF sCD27 levels using ELISA.

#### 3.2.3 Summary of Findings

Apart from Liu et al. (2018), all studies included in the systematic review reported significantly higher CSF sCD27 levels in patients with neuroinflammatory diseases compared to controls.

### 3.3 Risk of Bias Assessment

**FIGURE 2:**
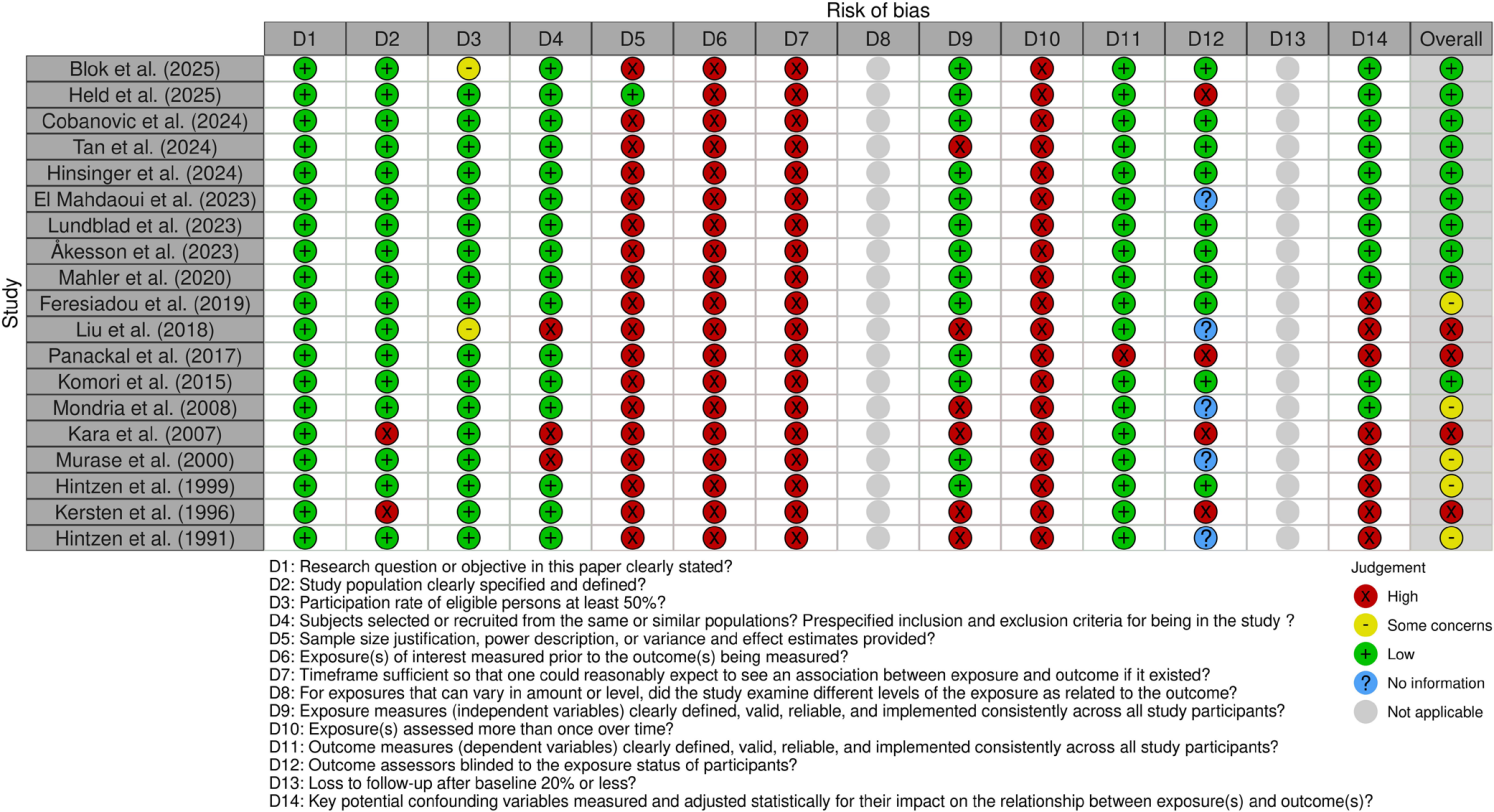
Risk of bias assessment. Risk of Bias assessment for included studies, where the first ten studies represent the studies included in the meta‐analysis. Risk of bias was evaluated using the National Heart, Lung, and Blood Institute (NHLBI) Quality Assessment Tool for Observational Cohort and Cross‐Sectional Studies. The figure was generated using the robvis tool, illustrating the overall quality and potential bias risk across studies.

The risk of bias assessment for all included studies is summarized in Figure 2.

Overall, eight studies were rated as having low risk of bias, seven as having some concerns, and four as having high risk of bias. Among the ten studies included in the meta‐analysis, one study (Liu et al. 2018) was rated as having high risk, five as having some concerns, and four as having low risk of bias.

In most studies, case populations were clearly defined and control groups were appropriately selected.

However, in the study by Feresiadou et al. (2019) which included the largest cohort, complete medical records were only available for half of the patient cohort. Thus, case ascertainment was limited, with the risk of misclassification.

Immunomodulatory treatment status, an important potential source of bias due to its possible effect on CSF sCD27 levels, was variably reported. In total, nine studies stated that patients were untreated at the time of sampling, three included both treated and untreated patients, one included only treated patients, and six did not report the treatment status. Among the studies included in the meta‐analysis, five included untreated patients only, two had mixed treatment status, and three did not report the treatment status, including Feresiadou et al. (2019).

Outcome assessment was generally robust, with appropriate sample handling and use of commercial or in‐house validated assays in most studies. However, a common concern was insufficient adjustment for potential confounders, particularly age and sex. Eight studies neither addressed nor adjusted for these variables, five of which were included in the meta‐analysis.

### 3.4 Descriptive Data

Table 2 summarizes the demographic characteristics and CSF sCD27 levels from the ten studies included in the meta‐analysis (Blok et al. 2025; Cobanovic et al. 2024; Feresiadou et al. 2019; Hintzen et al. 1999; Hintzen, van Lier, et al. 1991; Liu et al. 2018; Lundblad et al. 2023; Mahler et al. 2020; Mondria et al. 2008; Murase et al. 2000).

**TABLE 2:** Demographics and cerebrospinal fluid (CSF) soluble CD27 in studies included in the meta‐analysis.

	No. of studies	N	Mean age (range)	% Female	Mean CSF sCD27 in U/mL ± SD (*n*)	Mean CSF sCD27 in pg/mL ± SD (*n*)
Neuroinflammatory patients						
Disease groups						
MS	7	241	38.8 (15–68)	70	62.4 ± 81.7 (105)	1850.3 ± 3814.9 (136)
NMOSD	1	31	38.0 (10–59)	64.5	20.5 ± 14.7 (31)	NA
AE	1	24	NA	NA	NA	19032.0 ± 83480.7 (24)
PCNSL	1	13	63.2 (41–85)	30.8	123.5 ± 179.2 (13)	NA
Mixed inflammatory	3	376	48.4 (0–95)	51.7	83.0 ± 101.8 (38)	1376.0 ± 1561.0 (338)
Total	**10**	**685**	**44.7 (0–95)** ^**a**^	**55.6** ^**b**^	**63.9 ± 87.5 (187)**	**2356 ± 18 500 (498)**
Controls						
Control groups						
HC	2	79	32.6 (18–74)	55.7	NA	105.1 ± 99.8 (79)
SC	3	109	40.8 (19–85)	73.4	NA	188.4 ± 128,0.5 (109)
SAS	1	42	67.6 (45–85)	4.8	NA	280.0 ± 130.0 (42)
NIND	7	521	59.8 (1–88)	53.1	6.4 ± 6.5 (163)	68.7 ± 60.4 (358)
Total	**10**	**751**	**54.6 (1–88)** ^**c**^	**53.6** ^**c**^	**6.4 ± 6.5 (163)**	**110.9 ± 96.4 (588)**

*Note:* Data represent weighted means based on sample size when more than one study contributed to the estimate.

Abbreviations: AE, autoimmune encephalitis; CSF, cerebrospinal fluid; HC, healthy controls; MS, multiple sclerosis; *n*, number of participants per group; NA, not available; NIND, non‐inflammatory neurological disease; NMOSD, neuromyelitis optica spectrum disorder; PCNSL, primary central nervous system lymphoma; pg/mL, picogram per milliliter; SAS, spinal anesthesia subjects; sCD27, soluble CD27; SC, symptomatic controls; SD, standard deviation; U/mL, units per milliliter.

^a^ NA for 22 MS, 24 AE, 26 mixed inflammatory.

^b^ NA for 24 AE, 78 MS, 26 mixed inflammatory.

^c^ NA for 68 NIND.

The meta‐analysis comprised a total of 685 patients with neuroinflammatory diseases and 751 controls without neuroinflammatory conditions. Most patients were diagnosed with MS or categorized under IND. The majority of controls were patients categorized under NIND.

The weighted mean age was 44.7 years (range 0–95) for neuroinflammatory patients and 54.6 years (range 1–88) for controls. Female participants accounted for 55.6% of the patient group and 53.6% of the control group, calculated as weighted percentages.

Due to varying measurement units across studies, weighted mean CSF sCD27 levels were calculated separately in U/mL and pg/mL. Among neuroinflammatory patients, mean CSF sCD27 levels were 63.9 U/mL (SD 87.5) and 2356 pg/mL (SD 18500). In controls, mean levels were 6.4 U/mL (SD 6.5) and 110.9 pg/mL (SD 96.4). The notably large standard deviation in pg/mL was primarily driven by the study of Cobanovic et al. (2024), for which the mean and SD were estimated from median and IQR values. This study included a disease subgroup of patients with NMDA receptor autoimmune encephalitis, some of whom had concomitant herpes encephalitis or CNS lymphoma, leading to substantially elevated CSF sCD27 levels and consequently inflating the SD.

For studies with missing demographic data, those specific values were excluded from the weighted mean calculations (see Table 2 for details).

### 3.5 Meta‐Analysis

A meta‐analysis was conducted to quantitatively synthesize CSF sCD27 levels in neuroinflammatory diseases compared to controls across the ten included studies.

#### 3.5.1 Multivariate Random‐Effects Model

**FIGURE 3:**
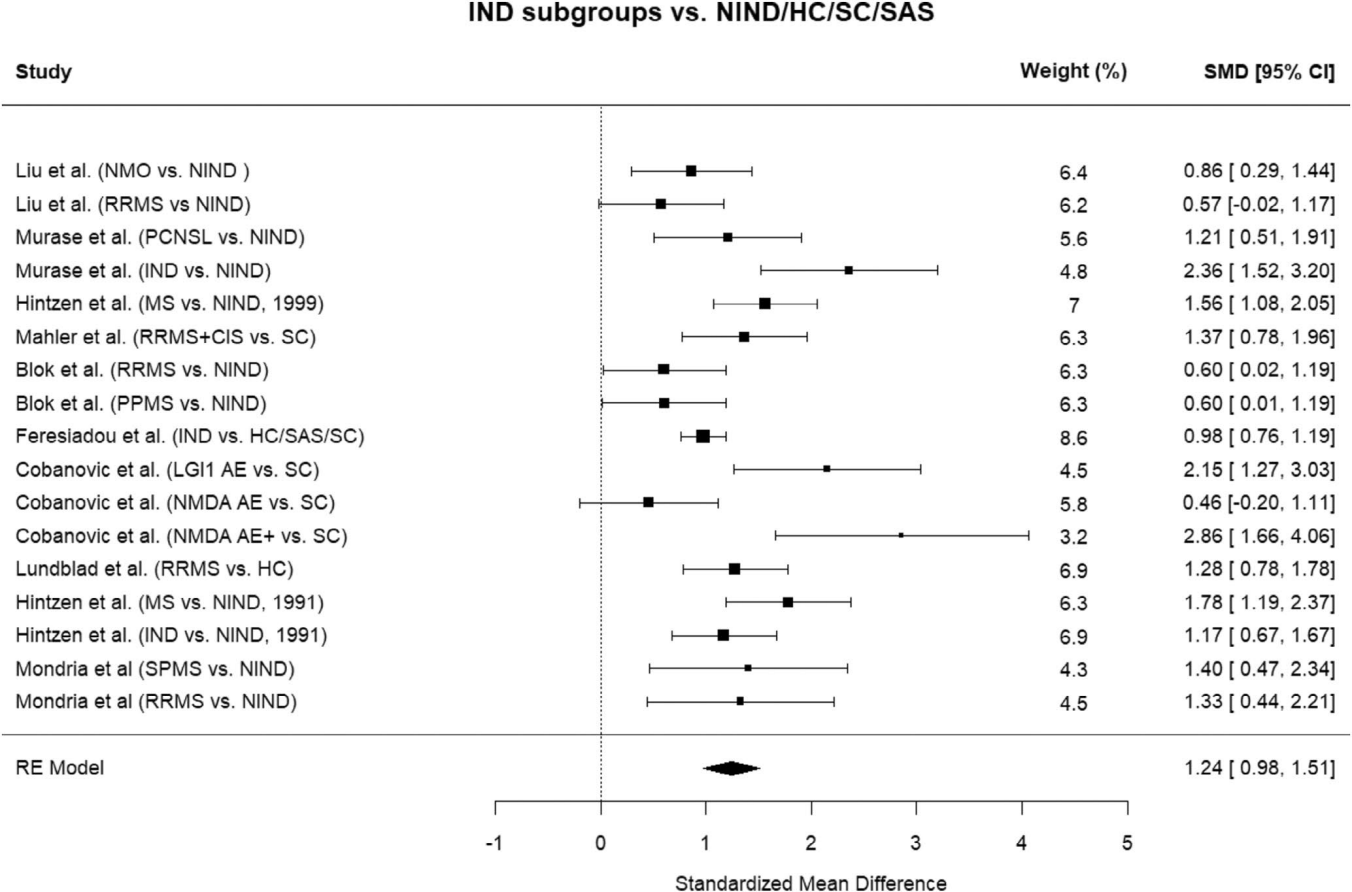
Multivariate meta‐analysis of cerebrospinal fluid (CSF) soluble CD27 levels. Forest plot of the multivariate meta‐analysis comparing cerebrospinal fluid soluble CD27 levels in patients with neuroinflammatory diseases versus controls. Standardized mean differences (SMDs) with 95% confidence intervals (CI) are shown. AE, autoimmune encephalitis; AE+, autoimmune encephalitis with additional neuroinflammatory diagnoses; CIS, clinically isolated syndrome; HC, healthy controls; IND, inflammatory neurological disease; MS, multiple sclerosis; NIND, non‐inflammatory neurological disease; NMO, neuromyelitis optica; PCNSL, primary central nervous system lymphoma; PPMS, primary progressive multiple sclerosis; RE, random effects; RRMS, relapsing–remitting multiple sclerosis; SAS, spinal anesthesia subjects; SC, symptomatic controls.

To maximize data utilization and account for correlations arising from shared control or disease groups within studies, a multivariate random‐effects model incorporating the variance–covariance matrix was applied. This analysis demonstrated substantial and significantly higher levels of CSF sCD27 in neuroinflammatory patients compared to controls (SMD = 1.24, 95% CI: 0.98–1.51, *p* < 0.0001) (Figure 3). Considerable heterogeneity was observed between studies (*Q* = 47.00, *p* < 0.0001; *I* ^2^ = 77.3%), supporting the use of a random‐effects approach. Sensitivity analysis excluding the study by Liu et al. (2018), which was rated as having high risk of bias, yielded results consistent with the primary analysis (data not shown).

#### 3.5.2 Collapsed Subgroup Meta‐Analysis

**FIGURE 4:**
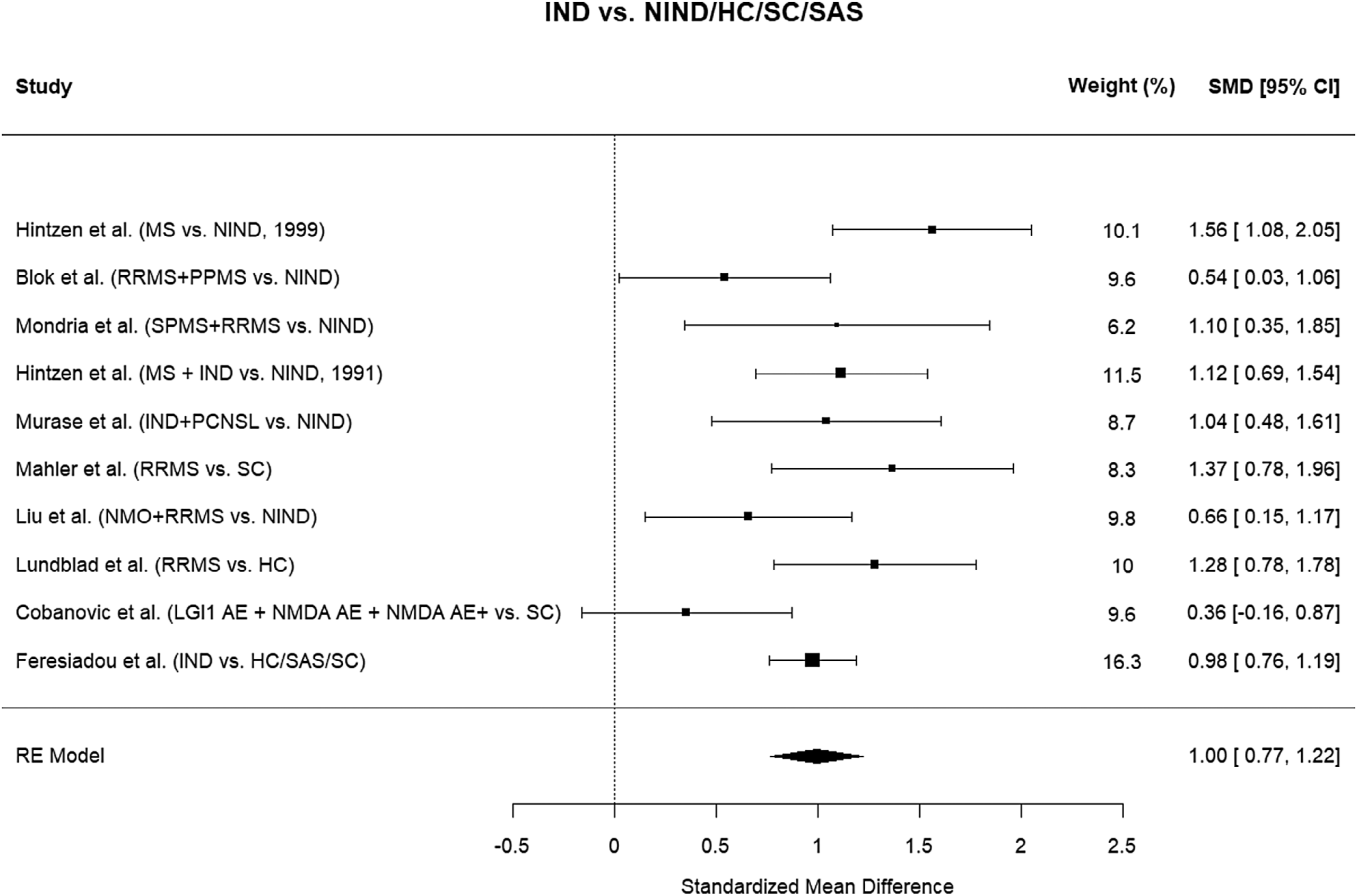
Meta‐analysis of cerebrospinal fluid (CSF) soluble CD27 using collapsed subgroups. Forest plot illustrating the standardized mean differences (SMD) and 95% confidence intervals (CI) from the random‐effects meta‐analysis using collapsed subgroups of neuroinflammatory patients and controls. This approach combines multiple subgroups within studies into a single group to provide an overall effect size estimate. AE, autoimmune encephalitis; AE+, autoimmune encephalitis with additional neuroinflammatory diagnoses; CIS, clinically isolated syndrome; HC, healthy controls; IND, inflammatory neurological disease; MS, multiple sclerosis; NIND, non‐inflammatory neurological disease; NMO, neuromyelitis optica; PCNSL, primary central nervous system lymphoma; PPMS, primary progressive multiple sclerosis; RE, random effects; RRMS, relapsing–remitting multiple sclerosis; SAS, spinal anesthesia subjects; SC, symptomatic controls.

As a complementary approach, we performed a random‐effects meta‐analysis after collapsing subgroups within studies to generate a single disease and control group for each study (Figure 4). This yielded a slightly smaller effect size (SMD = 1.00, 95% CI: 0.77–1.22, *p* < 0.0001). Between‐study heterogeneity was moderate (Q = 19.00, *p* = 0.0259; *I* ^2^ = 57.0%). Exclusion of the study by Cobanovic et al. (2024), which showed inflated variance due to data conversion, resulted in a modest increase in effect size (SMD = 1.06, 95% CI: 0.86–1.26, *p* < 0.0001) and a reduction of heterogeneity to a nonsignificant level (Q = 12.70, *p* = 0.1228; *I* ^2^ = 38.3%).

#### 3.5.3 Subgroup Meta‐Analysis

**FIGURE 5:**
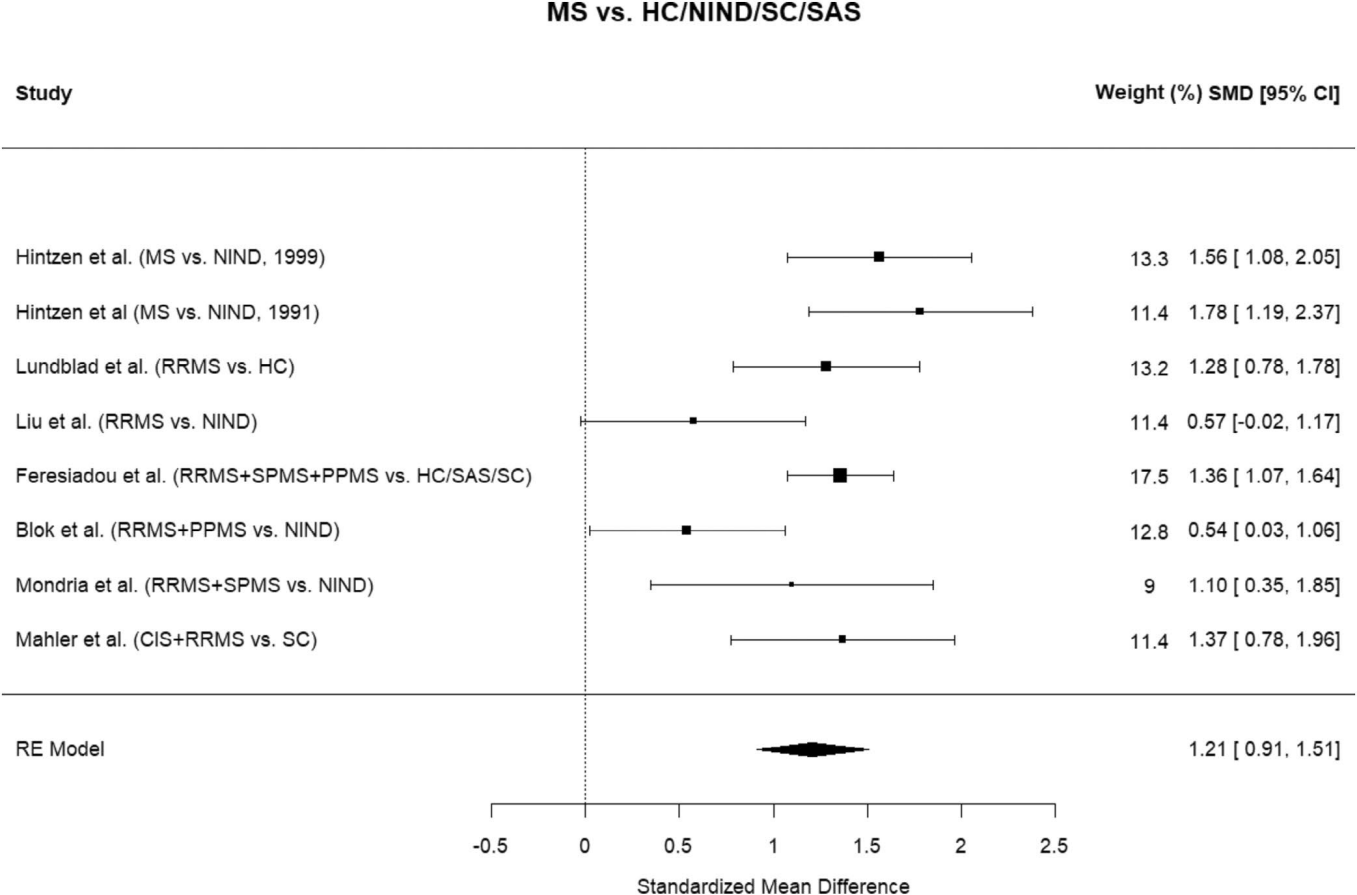
Meta‐analysis of cerebrospinal fluid (CSF) soluble CD27 in multiple sclerosis (MS) patients. Forest plot showing standardized mean differences (SMD) and 95% confidence intervals (CI) from the random‐effects meta‐analysis including only multiple sclerosis (MS) patients. Subgroups within each study were combined into a single MS group to estimate the overall effect size. CIS, clinically isolated syndrome; HC, healthy controls; MS, multiple sclerosis; NIND, non‐inflammatory neurological disease; PPMS, primary progressive multiple sclerosis; RE, random effects; RRMS, relapsing–remitting multiple sclerosis; SC, symptomatic controls.

To investigate potential differences in effect size across specific neuroinflammatory conditions, a subgroup meta‐analysis was performed including only studies reporting CSF sCD27 levels in MS patients, as this represented the largest subgroup. MS subgroups within individual studies were combined prior to analysis using the same random‐effects model (Figure 5). This yielded a large effect size comparable to the main analyses (SMD = 1.21, 95% CI: 0.91–1.51, *p* < 0.0001), with moderate heterogeneity (Q = 17.63, *p* = 0.0137; *I* ^2^ = 63.2%).

## 4 Discussion

This systematic review and meta‐analysis synthesized data from 19 studies investigating CSF levels of sCD27 in neuroinflammatory diseases. Of these, 10 studies provided sufficient quantitative data for meta‐analysis. Across analyses, CSF sCD27 was significantly higher in patients with neuroinflammatory conditions compared to controls, with standardized mean differences ranging from 1.00 to 1.24, indicating a large effect size. These findings support the role of CSF sCD27 as a robust marker of intrathecal adaptive immune‐mediated neuroinflammation.

sCD27 is a well‐established biomarker of adaptive immune activation. Since its identification by Hintzen, de Jong, et al. (1991); Hintzen, van Lier, et al. (1991), CSF sCD27 has consistently been associated with intrathecal inflammation in MS and other neuroinflammatory conditions, including NMOSD, CNS lymphoma, autoimmune encephalitis, and infectious CNS diseases (Cencioni et al. 2024; Cobanovic et al. 2024; Feresiadou et al. 2019; Hintzen et al. 1999; Komori et al. 2015; Murase et al. 2000).

Whether sCD27 primarily reflects T cell or B cell activation remains uncertain. Traditionally, sCD27 has been considered a marker of T cell activation, and recent evidence by Cencioni et al. (2024) supports this, demonstrating that sCD27 is predominantly produced by T cells, with limited contribution from B cells and plasmablasts. Notably, however, Cencioni et al. (2024) also found that sCD27 showed the strongest correlation with plasmablasts compared to other cell types in MS patients. Other studies also suggest a significant association between sCD27 and B cell activity in MS, particularly plasmablast/plasma cell differentiation and IgG production, supported by observed correlations with IgG index and soluble B cell maturation antigen (Agematsu et al. 1995; El Mahdaoui et al. 2023; Jacquot et al. 1997). However, the precise role of the CD70/CD27 interaction in B cell biology remains incompletely understood and warrants further investigation.

Despite these mechanistic uncertainties, the evidence from this systematic review and prior studies consistently demonstrates that CSF sCD27 levels are elevated in neuroinflammatory diseases compared to controls. This finding was consistent across all included studies except (Liu et al. 2018) which had methodological limitations. This reinforces the reliability and clinical relevance of CSF sCD27 as a biomarker for adaptive immune‐mediated neuroinflammation.

### 4.1 Limitations by Included Studies

A key limitation of the included studies is the heterogeneity in assay methods and measurement units used to quantify CSF sCD27. This variability likely contributes to differences in reported levels across studies and increases between‐study heterogeneity. It also complicates interpretation of what constitutes an elevated CSF sCD27 level, underscoring the need for standardized assays and reference values. Likewise, substantial heterogeneity in the definition of control‐groups exists for the NIND group. Although we have taken methodological measures to reduce risk of bias by the exclusion of neurodegenerative diseases with low‐grade inflammation (Parkinson's and Huntington's Disease), it is likely that heterogeneity remains in this control‐group.

On the other hand, the included studies may introduce spectrum bias since many of the studies only include patients from “extreme” sides of the clinical spectrum (e.g., non‐inflammatory controls and definite neuroinflammatory diseases), which may lead to overestimation of diagnostic accuracy, also in the results from our meta‐analysis.

This review included four proteomics‐based studies that detected CSF sCD27 which consistently reported significantly higher levels in neuroinflammatory diseases versus controls, expressed as fold changes (Åkesson et al. 2023; Held et al. 2025; Hinsinger et al. 2024; Tan et al. 2024). Notably, several proteomics studies were excluded because they failed to detect CSF sCD27 altogether. This discrepancy may stem from differences in platform sensitivity, protein coverage, or technical limitations inherent to proteomic assays, such as low abundance of sCD27 relative to detection thresholds or issues with protein extraction and identification methods.

Another important limitation is that most studies employed retrospective designs, leading to inconsistent reporting of treatment status and missing clinical data in some cohorts. For example, Feresiadou et al. (2019), which contained the largest cohort, only had complete medical data for half of the cases. This raises concerns about the accurate classification of neuroinflammatory status within the case cohort. Additionally, the inclusion of treated patients or mixed treatment populations may have introduced a downward bias in CSF sCD27 levels, as immunomodulatory therapies are known to attenuate inflammatory activity and hence reduce the levels of inflammation markers (Cross et al. 2024; Konen et al. 2020; Mellergård et al. 2010; Romme Christensen et al. 2019).

Moreover, several studies did not adequately control for key confounders such as age, sex, and blood‐CSF‐barrier integrity (e.g., albumin ratio). Tigchelaar et al. (2024) conducted a large study in a neurologically healthy surgical population and found modest positive correlations between CSF sCD27 and age (*r* = 0.32), albumin ratio (*r* = 0.31), and plasma sCD27 (*r* = 0.28). This contrasts with earlier smaller studies, which found no significant correlations (Blok et al. 2025; Cobanovic et al. 2024; Feresiadou et al. 2019; Hintzen, van Lier, et al. 1991; Kersten et al. 1996; Komori et al. 2015; Lundblad et al. 2023; Mahler et al. 2020; Tan et al. 2024). Tigchelaar et al. (2024) suggest that previous null findings may reflect limited sample sizes, narrow age ranges, and the use of symptomatic or neuroinflammatory controls rather than healthy populations. Importantly, several neuroinflammatory diseases have varying degrees of damage to the blood‐CSF‐barrier, and sCD27 levels in blood are approximately 30‐fold higher than CSF (Tigchelaar et al. 2024). Therefore, CSF sCD27 levels may be increased in these disorders due to diffusion of sCD27 over a damaged blood‐CSF‐barrier, and not necessarily reflect intrathecal synthesis. Thus, the influence of demographic factors and blood‐CSF‐barrier function on CSF sCD27 remains incompletely understood, complicating interpretation of studies that do not adjust for these confounders.

### 4.2 Diagnostic Potential

Most studies report strong diagnostic performance of CSF sCD27 in distinguishing neuroinflammatory diseases from non‐inflammatory neurological diseases and controls. Among the studies reporting AUC values, all but one demonstrated excellent discrimination, with AUCs consistently exceeding 0.85 (Åkesson et al. 2023; Cobanovic et al. 2024; Feresiadou et al. 2019; Hinsinger et al. 2024; Kersten et al. 1996; Komori et al. 2015; Mahler et al. 2020). Notably, Feresiadou et al. (2019), the largest cohort, reported an AUC of 0.89 when comparing IND with NIND. They also found an AUC of 0.84 when distinguishing infectious CNS disease from sterile inflammation, suggesting that CSF sCD27 levels may be even higher in infectious conditions than in non‐infectious neuroinflammation. This aligns with findings from Cobanovic et al. (2024), who observed markedly elevated CSF sCD27 levels in autoimmune encephalitis patients with preceding or concurrent herpes encephalitis. In contrast, Tan et al. (2024) reported a lower AUC of 0.77, which may reflect the small size of the control group and unclear neuroinflammatory status due to missing data.

Overall, the consistently high AUC values reported across studies support the diagnostic utility of CSF sCD27 for identifying neuroinflammatory diseases. Based on its strong ability to identify adaptive immune‐mediated neuroinflammation, CSF sCD27 may be developed further for implementation in clinical practice as a diagnostic biomarker for diagnosis of neuroinflammatory diseases, complementary to existing CSF routine diagnostic biomarkers like cell count, IgG index, and oligoclonal bands. Development of a diagnostic assay with short analysis time may enable early and accurate identification of neuroinflammation and thereby facilitate reliable rule‐out of adaptive immune‐mediated neuroinflammation. Further validation across a wider range of neuroinflammatory conditions in well‐characterized cohorts, assessment of association with age, albumin ratio, and plasma levels, establishment of cut‐off levels, and development of diagnostic algorithms is needed before clinical implementation.

### 4.3 Review Limitations

This review has several limitations that may affect the interpretation and generalizability of the findings. First, eleven potentially relevant studies were excluded at the full‐text screening stage due to inappropriate or missing control groups, highlighting a notable gap in the literature. The exclusion of these studies may introduce selection bias and limit the representativeness of the meta‐analysis. Second, nine studies were excluded from the meta‐analysis due to insufficient quantitative data, despite reporting similar trends of elevated CSF sCD27. Their inclusion could have increased the statistical power and robustness of the meta‐analytic estimates, so their absence potentially weakens the overall conclusions. Third, the relatively small number of studies included in the meta‐analysis prevented formal assessment of publication bias. This leaves open the possibility that positive results may be overrepresented in the literature.

Regarding study quality, only eight included studies were rated as low risk of bias, with seven having some concerns and four rated as high risk. Although sensitivity analysis excluding the high‐risk study (Liu et al. 2018) in the meta‐analysis did not significantly alter the results, residual bias in included studies could still influence effect size estimates.

Additionally, several studies reported medians and IQR rather than means and SD, necessitating conversion using estimation methods. While these methods are validated (Luo et al. 2018; Wan et al. 2014), they may produce imprecise estimates, especially for biomarkers like sCD27 with right‐skewed distributions. This was evident in the inflated variance observed in Cobanovic et al. (2024), potentially affecting heterogeneity and effect size calculations.

Finally, considerable between‐study heterogeneity was observed, likely reflecting variations in study design, populations, assay methods, and reporting. This heterogeneity reduces the precision and generalizability of the meta‐analytic findings and underscores the need for standardized methodologies in future research.

## 5 Conclusions and Future Directions

To our knowledge, this is the first systematic review and meta‐analysis examining CSF sCD27 as a biomarker for adaptive immune‐mediated neuroinflammation. The findings of the review and meta‐analysis demonstrate that CSF sCD27 is markedly elevated in the neuroinflammatory diseases studied compared to controls, supporting its role as a robust marker of adaptive immune‐mediated neuroinflammation. Despite methodological variability across studies, the overall effect size was large and consistent. To fully realize the diagnostic potential of CSF sCD27, future research should prioritize standardized assay methods and reporting units, along with consistent documentation of demographic, clinical, and treatment data. Larger prospective studies across a broader spectrum of neuroinflammatory diseases are essential to validate these findings. Together, these steps will strengthen the evidence base and support the integration of CSF sCD27 into diagnostic protocols for adaptive immune‐mediated neuroinflammation.

## Author Contributions

**Nadia Damholt Savino:** conceptualization, methodology, formal analysis, investigation, visualization, writing – review and editing, writing – original draft. **Amanda Marie Lund Christiansen:** investigation, writing – review and editing. **Jeppe Romme Christensen:** conceptualization, methodology, writing – review and editing, investigation. **Finn Sellebjerg:** conceptualization, methodology, writing – review and editing. **Sahla El Mahdaoui:** methodology, writing – review and editing. **Malene Bredahl Hansen:** investigation, writing – review and editing.

## Funding

Nadia Damholt Savino and Jeppe Romme Christensen were funded by a grant from the Ellab foundation. Amanda Marie Lund Christiansen was funded by the Rigshospitalet's Research Fund. Finn Sellebjerg holds a professorship at the Department of Clinical Medicine, University of Copenhagen sponsored by the Danish Multiple Sclerosis Society.

## Conflicts of Interest

Nadia Damholt Savino has received non‐financial support for conference participation from Merck. SEM has received speaker honoraria and non‐financial support for conference participation from Merck and honoraria from Novartis for contribution to educational material. Finn Sellebjerg has served on scientific advisory boards for, served as consultant for, received support for congress participation, or received speaker honoraria from Biogen, Lundbeck, Merck, Neuraxpharm, Novartis, Roche, and Sanofi. His laboratory has received research support from Biogen, Merck, Novartis, Roche, and Sanofi. Finn Sellebjerg is section editor on Multiple Sclerosis and Related Disorders. Malene Bredahl Hansen, Amanda Marie Lund Christiansen, and Jeppe Romme Christensen declare no conflicts of interest.

## Supporting information


**Table S1:** Study characteristics for all included studies.

## Data Availability

The data that support the findings of this study are available from the corresponding author upon reasonable request.
