# Supplementary material for: Cerebrospinal Fluid sCD27 as a Biomarker of Neuroinflammatory Disease: A Systematic Review and Meta‐Analysis
Source: J Neurochem. 2026 Apr 28;170:e70451. doi: 10.1111/jnc.70451 (PMC13123749; doi:10.1111/jnc.70451)
Supplement: Supplementary file 1 — Table S1: Study characteristics for all included studies. [file JNC-170-e70451-s001.pdf]

Title: Cerebrospinal Fluid sCD27 as a Biomarker of Neuroinflammatory Disease: A Systematic Review and Meta-Analysis

Authors:

Nadia Damholt Savino[1,2], Malene Bredahl Hansen[1], Amanda Marie Lund Christiansen[1,2], Sahla El Mahdaoui[1], Finn Sellebjerg[1,2], Jeppe Romme Christensen[1]

Affiliations:

[1] Danish Multiple Sclerosis Center, Copenhagen University Hospital – Rigshospitalet, Glostrup, Denmark

[2] Department of Health and Medical Sciences, University of Copenhagen, Copenhagen, Denmark

Correspondence:

Please address proofs and reprint requests to:

Nadia Damholt Savino, Danish Multiple Sclerosis Center, Copenhagen University Hospital – Glostrup, Valdemar Hansens Vej 13, 2600 Glostrup, Denmark.

Email: nadia.damholt.savino@regionh.dk

Telephone: +45 53342722

Protocol

Systematic review: CD27 and neuroinflammatory disease

Research question:

Research question was based on methods described in article by Luijendijk (2021):

Question Components	Components in your review
**P – Patient or Population**<br>Describe the most important characteristics of the patient.	Patients with symptoms of neurological disease
**I – Index test (positive result)**	High levels of sCD27 in CSF
**C – Comparison; index test (negative result)**	Low levels of sCD27 in CSF
**O – Outcome**	Neuroinflammation*
**Your research question: Can high levels of sCD27 predict neuroinflammation in all neuroinflammatory diseases?**	

*Neuroinflammation determined by standardised diagnostic tests (reference tests), e.g. MRI, cerebrospinal fluid biomarkers (cell count, IgG index, albuminratio, oligoclonal bands), auto-antibodies, neurological examination, etc.

Methods:

Outcome:

- Levels of CSF sCD27 in patients with neuroinflammatory disease compared to controls with no neuroinflammatory disease

Additional outcomes:

- CSF sCD27 with cell count, IgG index, albumin ratio, oligoclonal bands

Inclusion criteria:

- Full text article in English
- Observational design (cohort, case-control, cross-sectional)
- Reporting of sCD27 levels in CSF in neuroinflammatory disease compared to control group with no neuroinflammatory disease
- Availability of data on mean or median CSF sCD27 levels, or AUC with or without sensitivity and specificity, or fold change values
- Studies published in peer-reviewed journals

Exclusion criteria:

- No reporting of quantitative data of CSF sCD27 levels
- No full text available
- Animal studies or non-peer-reviewed sources
- No control group consisting of patients with no neuroinflammatory disease

Search strategy:

- Databases: Pubmed, Embase, Scopus,

Risk of bias assessment:

- Robvis tool (McGuinness & Higgins, 2021)
- National Heart, Lung, and Blood Institute (NHLBI) quality assessment tool for case-control studies (NHLBI, n.d.)

Data extraction:

- Study characteristics: first author, publication year, number of patients per disease group, method used for sCD27 detection, population demographics (sex, age), immunomodulatory treatment prior to sample collection
- Primary outcome measures: levels of sCD27 (e.g. mean, median, standard deviation, range, fold change), sensitivity, specificity, AUC, or applied cut-off values to define test positivity
- Additional outcome measures (if available): correlations with cell count, IgG index, albumin ratio, oligoclonal bands

Statistical methods:

- Pooled standardized mean difference (SMD)
- Effect size calculation
- Publication bias: funnel plots and Eggers test if possible
- Subgroup analyses

- Sensitivity analysis

References:

Luijendijk, H. J. (2021). How to create PICO questions about diagnostic tests. *BMJ Evidence-Based Medicine*, *26*(4), 155–157. https://doi.org/10.1136/bmjebm-2021-111676

McGuinness, L. A., & Higgins, J. P. T. (2021). Risk-of-bias VISualization (robvis): An R package and Shiny web app for visualizing risk-of-bias assessments. *Research Synthesis Methods*, *12*(1), 55–61. https://doi.org/10.1002/jrsm.1411

NHLBI. (n.d.). *Study quality assessment tools*. National Institutes of Health. Retrieved October 14, 2025, from https://www.nhlbi.nih.gov/health-topics/study-quality-assessment-tools

Supplementary Table 1. Study characteristics for all included studies.

Study characteristics			Demographics and Methods				Outcome		
**Author & Year**	**Study design**	**Cohort: n**	**Sex (%female)**	**Age (mean or median), years**	**Immunomodulatory treatment**	**sCD27 detection method**	**CSF sCD27 level**	**AUC**	**Applied cut-off value?**
Liu et al. (2018)	Retrospective	NMOSD: 31<br>RRMS: 23<br>NIND: 22	NMOSD: 64.5<br>RRMS: 43.5<br>NIND: 63.6	NMOSD: 38<br>RRMS: 38<br>NIND: 35	No immunosuppressive treatment at sample time<br>Samples taken during a relapse	ELISA (Thermo Fischer Scientific)<br><br>U/mL	sCD27 mean:<br>NMOSD: 20.46<br>RRMS: 13.24<br>NIND: 10.35<br><br>Significance:<br>NMOSD vs. NIND: S<br>NMOSD vs. MS: NS<br>MS vs. NIND: NS	NR	NR
Murase et al. (2000)	Unclear from article	PCNSL: 13<br>HC: 50*<br>OBT: 30<br>NIND: 25**<br>IND: 12<br><br>*only serum samples, excluded from this study<br><br>**includes patients with CNS lupus and parkinson's, NIND group will therefore be excluded in this study	PCNSL: 30.8<br>OBT: 40.0<br>NIND*: 48.0<br>IND: 33.3<br><br>*Excluded	PCNSL: 63.2<br>OBT: 35.5<br>NIND*: 56.5<br>IND: 39.0<br><br>*Excluded	PCNSL:<br>All: % corticosteroid within one week<br>10: during treatment<br>4: during remission<br>12: treatment naïve<br><br>OBT + IND: treatment naive	Sandwich ELISA in-house developed<br><br>U/mL	sCD27 mean:<br>PCNSL (26 samples): 123.5<br>OBT: 4.2<br>NIND: 4.4<br>IND: 74.4<br><br>Significance:<br>*PCNSL vs. OBT+NIND: S*<br>*PCNSL vs. IND: NS*	AUC: NR<br><br>PCNSL+IND vs. OBT+NIND:<br>Sensitivity 100%<br>Specificity 83.6%	15 U/mL
Hintzen et al. (1991)	Retrospective	MS: 19<br>*-RRMS:16*<br>*-PPMS: 3*<br>NIND: 56<br>IND: 26	MS: 63.2<br>NIND: NR<br>IND: NR	MS: 37<br>NIND: NR<br>IND: NR	No treatment within 6 months prior to sampling	ELISA (T Cell Sciences, Cambridge)<br><br>U/mL	sCD27 median:<br>MS: 63<br>NIND: 7<br>IND: 44<br><br>Significance:<br>*MS +IND vs. NIND: S*	NR	NR
Mondria et al. (2008)	Prospective	SPMS+ BMT:14*<br>RRMS: 12<br>NIND: 17	NR	NR	SPMS: 10 samples pre BMT, 8 samples post BMT	ELISA (CLB, Amsterdam)	sCD27 median:<br>SPMS preBMT: 112	NR	NR

		 *only 10 CSF samples pretransplantation			RRMS: NR<br>NIND: NR	U/mL	SPMS postBMT: 55<br>NIND: 4.0<br>RRMS: 54.5<br><br>Significance:<br>*PreBMT vs. postBMT: S*<br>*postBMT vs. RRMS: NS*<br>*SPMS vs. NIND: S*<br>*SPMS vs. RRMS: S*		
Hintzen RQ, Paty D and Oger J (1999)	Retrospective	HAM/TSP: 8*<br>HTLV-I carriers: 8*<br>MS: 41<br>*-RRMS: 30*<br>*-SPMS: 11*<br>NIND: 43<br><br>*Excluded from this study	HAM/TSP: 50.0<br>HTLV-I carriers: 75.0<br>MS: 56.1<br>NIND: 55.8	HAM/TSP: 58<br>HTLV-I carriers: 47<br>MS: 39<br>NIND: 40	NR	ELISA (CLB, Amsterdam)<br><br>U/mL	sCD27 mean:<br>HAM/TSP: 143<br>HTLV-I carriers: 19<br>NIND: 3<br>MS: 46<br><br>Significance:<br>*MS vs. NIND: S*<br>*RRMS vs. SPMS: NS*	NR	16 U/mL
Lundblad et al. (2023)	Prospective	RRMS AHSCT+: 45<br>HC: 32	RRMS: 64.4<br>HC: 46.9	RRMS: 30<br>HC: 22	CSF samples are taken pre – and post AHSCT.<br><br>Pre AHSCT:<br>DMT+: 35<br>DMT-: 10	ELISA (R&D systems)<br><br>pg/mL	sCD27 median:<br>RRMS<br>*-Pre AHSCT: 352*<br>*-Post AHSCT (1y): 143*<br>*-Post AHSCT (2y): 120*<br>HC: 63<br><br>Significance:<br>*RRMS pre AHSCT vs HC: S*<br>*RRMS pre vs. post AHSCT: S*	NR	NR
Feresiadou et al. (2019)	Retrospective	IND: 338<br>NIND: 338<br>Controls: 127<br>*-HC:47*<br>*-SC: 38*<br>*-SAS: 42 (urologic surgery)*	Controls:47,2<br>NIND: 49,7<br>IND: 52,3	Available for all subgroups in article table	NR	ELISA (R&D systems)<br><br>pg/mL	sCD27 median:<br>Controls: 64<br>*-HC 16*<br>*-SC 13*<br>*-SAS 252*<br>NIND 58<br>IND 740	IND vs NIND:<br>*AUC = 0.89 (S)*<br>*Sensitivity 74%*<br>*Specificity93%*<br><br>Infectious disease vs	250 pg /mL (IND vs NIND)<br><br>2500 pg /mL

							Significance:<br>*NIND vs IND: S*<br>*IND vs. controls: S*	sterile inflammation:<br>*AUC= 0.84 (S)*<br>*Sensitivity: 40%*<br>*Specificitet: 96%*	(Infectious disease vs. sterile inflammation)
Cobanovic et al. (2024)	Retrospective	NMDA AE: 21<br>*-untreated: 12*<br>NMDA AE+: 5<br>*-untreated: 4**<br>LGI1 AE: 14<br>*-untreated: 8*<br>SC: 37<br><br>*3 herpes encephalitis, 1 CNS lymphoma	NMDA AE: 57,1<br>NMDA AE+: 20<br>LGI1 AE: 35,7<br>SC: 73,0	NMDA AE: 26<br>NMDA AE+: 67<br>LGI1 AE: 63<br>SC: 40	A subgroup of untreated patients is used in analysation of sCD27	ELISA (Human Magnetic Luminex Assay, biplex, R&D)<br><br>pg/mL	sCD27 median in untreated subgroups:<br>LGI1 AE: 551<br>NMDA AE: 1571<br>NMDA+ AE: 19988<br>SC: 250<br><br>Significance:<br>*SC vs. NMDA AE:S*<br>*SC vs. LGI1 AE: S*<br>*LGI AE vs. NMDA AE: S*<br>*Untreated AE vs. treated AE: NS*	Untreated AE vs. SC:<br>*AUC = 0.97 (S)*<br>*Sensitivity: 100%*<br>*Specificity: 73%*	304 pg /mL (untreated AE vs. SC)
Mahler et al. (2020)	Retrospective	Treated MS: 13<br>*-NZB: 6*<br>*-AZB: 7*<br>Untreated MS : 22<br>*-CIS: 6*<br>*-RRMS: 16*<br>SC: 34	*Untreated MS:* 81.8<br>Treated MS: 69.2<br>SC: 70.6	Untreated MS:<br>-CIS: 30<br>-RRMS: 31<br>Treated MS:<br>-NZB: 39<br>- AZB: 42<br>SC: 36	MS patients are divided in a treated and untreated group	ELISA (Human Magnetic Luminex Assay, R&D)<br><br>pg/mL	sCD27 median:<br>Treated MS:732<br>Untreated MS: 2460<br>SC: 270<br><br>*sCD27 mean*:*<br>Treated MS: 985<br>Untreated MS: 3360<br>SC: 285<br><br>*S between all groups	Untreated MS vs SC:<br>*AUC = 0.93 (S)*	NR
Blok et al. (2025)	Retrospective	PPMS:104<br>RRMS: 38<br>AD: 22<br>NMOSD 10*<br>MOGAD 10*<br><br>*Excluded from this study because	PPMS: 52.0<br>RRMS: 71.1<br>AD: 72.7	RRMS: 39.7<br>PPMS: 49.3<br>AD: 64.0	1 RRMS + 1 PPMS had corticosteroids within 4 weeks of CSF sample, otherwise treatment naive	ECLIA, MSD U-plex, triplex with sCD27, sBCMA and CHI3L1<br><br>pg/mL	sCD27 mean:<br>PPMS (27 CSF samples): 2444<br>RRMS (29 CSF samples): 2893<br>AD (20 CSF samples): 213	NR	NR

		of only serum samples					Exact data obtained from corresponding author via email		
Komori et al. (2015)	Prospective	Cohort A+B:<br>*PPMS 81*<br>*SPMS 74*<br>*RRMS 121*<br>*HC 8*<br>*NIND 57*<br>*OIND 45*	Cohort A+B:<br>PPMS: 44.4<br>SPMS: 58.1<br>RRMS: 57.0<br>HC: 50.0<br>NIND: 70.2<br>OIND: 33.3	Cohort A+B:<br>PPMS: 55<br>SPMS: 54<br>RRMS: 38<br>HC: 38<br>NIND: 51<br>OIND: 50	No immunomodulatory treatment min. 3 months prior to CSF collection (exception of a few OIND, not further specified)	ECLIA, MSD, In-House developed (Rockville, MD) and antibodies from Sanquin (Amsterdam)<br><br>U/mL	sCD27 median: Exact result can't be read from figure 4, but significantly higher levels in RRMS+PPMS+ SPMS+OIND vs HC+ NIND<br><br>Exact data could not be obtained after contacting author	(OIND+PPMS+ RRMS+SPMS) vs. (HC+NIND)<br>*AUC = 0.97*<br><br>(S)	NR
Kersten et al. (1996)	Prospective	5 groups:<br>***1: 50 SC***<br>*2*: 70 children with lymphoid malignancies examined for CNS involvement*<br>*3*: 47 ALL (symptoms suspected of meningeal localization)*<br>***4: 4 PCNSL***<br>*5*: 8 myeloid leukaemia + 24 solid tumors suspected of meningeal metastases*<br><br>*Excluded from this study because of systemic inflammation	NR	NR	NR (only info regarding 1 PCNSL patient: 1 sample pretreatment and 1 sample posttreatment)	ELISA In-House developed: Sandwich ELISA<br><br>U/mL	sCD27 median:<br>*SC: 1.66**<br>*PCNSL: 63**<br><br>**calculated from figure 2, as no exact results are given*<br><br>Exact data could not be obtained after contacting author	AUC NA for SC and PCNSL<br><br>Meningeal involvement vs. no meningeal involvement:<br>*Gr. 2:*<br>*AUC = 0.99 (S)*<br>*Sensitivity: 83%*<br>*Specificity: 96%*<br>*Gr. 3:*<br>*AUC= 0.95 (S)*<br>*Sensitivity: 100%*<br>*Specificity: 82%*	10 U/mL
El Mahdaoui et al. (2023)	Retrospective	RRMS: 40*<br>SC: 9*<br><br>*17 of RRMS patients and 8 of SC are used in Mahler et al	RRMS: 77.5<br>SC: 35.7	RRMS: 34<br>SC: 36	Treatment naive RRMS patients	ECLIA, MSD U-PLEX Human assay (duplex with sCD27 and sBCMA),<br><br>pg/mL	sCD27 median:<br>RRMS: 1020<br>SC: 130	NR	NR

Kara et al. (2007)	Retrospective	30 cases<br>*-ALL: 18*<br>*-NHL: 7*<br>*-AML: 5*<br>NIND: 5	NR	NR	CSF samples obtained for diagnosis or upon therapeutic intrathecal administration of cytotoxic drugs. Treatment status not further specified	ELISA (PeliKine Compact Human Soluble CD27 kit, Research Diagnostics Inc., Concord)<br><br>pg/mL	sCD27 mean:<br>In case group overall:<br>*-LI + (6): 126.44**<br>*-LI - (24): 68.05**<br>NIND: no exact value, in article written below cutoff value (350)<br><br>*exact numbers calculated from table 1, exact data could not be obtained from author	NR	NR
Panackal et al. (2017)	Prospective	Cryptococcal spinal arachnoiditis (IND): 6<br>HC: 11	IND: 16.7<br>HC: NR	IND: 49,5<br>HC: NR	All patients were treated with antifungal therapy at sampling<br><br>Samples pre – and post immunomodulatory treatment	Not clearly described in article<br><br>U/mL	sCD27 median:<br>IND: 153*<br>HC: 8*<br><br>*Read from figure 3, exact data could not be obtained from author	NR	NR
Åkesson et al. (2023)	Prospective	<u>Discovery cohort:</u><br>*MS: 92*<br>*-RRMS: 30*<br>*-CIS: 62*<br>*HC: 23*<br><br><u>Replication cohort:</u><br>*MS: 51*<br>*-RRMS: 30*<br>*-CIS: 21*<br>*HC: 20*	<u>Discovery cohort:</u><br>*MS: 72.8*<br>*HC: 78.3*<br><br><u>Replication cohort:</u><br>*MS: 76.5*<br>*HC: 50.0*	<u>Discovery cohort:</u><br>*MS: 31*<br>*HC: 32*<br><br><u>Replication cohort:</u><br>*MS: 32*<br>*HC: 30*	<u>Discovery cohort:</u><br>DMT within 3 m before baseline: 5 +, 87 -<br>Steroid treatment within 3 m before baseline: 9 +, 83 -<br><br><u>Replication cohort:</u><br>DMT within 3 m before baseline: 0 +, 51 -<br>Steroid treatment within 3 m before baseline: 2 +, 49 -	Olink Explore Platform using Proximity Extension Assay (PEA) technology combined with Next Generation Sequencing (NGS)	<u>Discovery cohort:</u><br>Log2FC*=2.77 (S)<br><br><u>Replication cohort:</u><br>Log2FC* =2.73 (S)<br><br>*MS vs. HC	<u>Discovery cohort:</u><br>*AUC=0.97* (S)*<br><br><u>Replication cohort:</u><br>*AUC=0.87* (S)*<br><br>*MS vs. HC	NR
Hinsinger et al. (2024)	Retrospective	<u>Discovery cohort:</u><br>*SC: 10*<br>*SC-CIS: 10*<br>*FC-CIS: 10*	<u>Verification cohort:</u><br>*SC: 80*<br>*SC-CIS: 73.3*	<u>Verification cohort:</u><br>*SC: 38.3*<br>*SC-CIS: 35.2*<br>*FC-CIS: 33.3*	NR	Label free and targeted quantitative proteomics	<u>Verification cohort:</u><br>sCD27 FC (S):	<u>Verification cohort:</u><br>AUC:	NR

		RRMS: 10<br><br>Qualification cohort:<br>*SC: 10*<br>*SC-CIS: 10*<br>*FC-CIS: 10*<br>*RRMS: 10*<br>*PPMS: 10*<br>*IND: 10*<br><br>Verification cohort:<br>*SC: 30*<br>*SC-CIS: 15*<br>*FC-CIS: 15*<br>*RRMS: 30*<br>*PPMS: 14*<br>*IND: 13*<br>*ION: 15*<br>*NIND: 13*<br>*PIND: 14*	*FC-CIS: 93.3*<br>*RRMS: 76.7*<br>*PPMS: 42.9*<br>*IND: 23.1*<br>*ION: 86.7*<br>*NIND: 46.2*<br>*PINDC: 53.8*	*RRMS: 38.2*<br>*PPMS: 46.6*<br>*IND: 46.4*<br>*ION: 31.8*<br>*NIND: 40.8*<br>*PIND: 56.2*		Specific sCD27 quantification in VC: ELISA, U-PLEX Human assay, MSD	NIND vs SC: 1.32<br>PIND vs. SC: 1.35<br>IND vs. SC: 3.66<br>RRMS vs SC: 10.64<br>PPMS vs. SC: 5.16<br>RRMS vs. IND: 2.91<br>RRMS vs. NIND: 8.06<br><br>CD27 levels measured by ELISA in 14 SC, 20 RRMS, and 9 PPMS → result cannot be read from graph in figure 4, but S difference in SC vs. RRMS and PPMS	*SC vs RRMS: 0.98 (S)*<br>*MS* vs IND: 0.76 (S)*<br>*(MS*+IND) vs. (PIND+NIND): 0.98 (S)*<br><br>*All disease stages of MS	
Held et al. (2024)	Retrospective	Discovery cohort:<br>*RRMS: 29*<br>*PPMS: 30*<br>*LNB: 8*<br>*NC: 20*<br><br>Verification cohort:<br>*RRMS: 10*<br>*PPMS: 10*<br>*NC: 8*	Discovery cohort:<br>*RRMS: 76.7*<br>*PPMS: 66.7*<br>*LNB: 37.5*<br>*NC: 75.0*<br><br>Verification cohort:<br>*RRMS: 50.0*<br>*PPMS: 50.0*<br>*NC: 50.0*	Discovery cohort:<br>*RRMS: 44*<br>*PPMS: 44*<br>*LNB: 44.5*<br>*NC: 48.5*<br><br>Verification cohort:<br>*RRMS: 51*<br>*PPMS: 53*<br>*NC: 52*	MS patients were treatment naive	Targeted proteomics (proximity extension assay), Olink platform	Discovery + verification cohort:<br>sCD27 log2FC: MS vs. NC: 2.88 (S)	NR	NR
Tan et al. (2024)	Retrospective	Cohort 1:<br>*RRMS: 25*<br>*SPMS: 25*<br>*PPMS: 8*<br>*NIND: 23*<br><br>Cohort 2: Prospective MS cohort (not included in this study, only serum	Cohort 1:<br>*RRMS: 40.0*<br>*SPMS: 48.0*<br>*PPMS: 37.5*<br>*NIND: 56.5*	Cohort 1:<br>*RRMS: 39.5*<br>*SPMS: 48.1*<br>*PPMS: 54.4*<br>*NIND: 45.5*	No DMT at inclusion	Targeted proteomics (proximity extension assay), Olink platform, Proseek multiplex immunooncology panel	sCD27 NPX:<br>NIND: 4.65<br>MS: 6.42<br>(S)<br><br>*FC*: 3,42*<br><br>**Calculated*	MS (58) vs. NIND (23):<br>*AUC =0.77 (S)*	NR

		samples and no control group)							

Key data extracted from each study. ***Abbrevations:*** *AD, Alzheimer's disease; AE, autoimmune encephalitis; AE+, autoimmune encephalitis with additional neuroinflammatory diagnoses; AHSCT, autologous hematopoietic stem cell transplantation; ALL, acute lymphoblastic leukaemia; AML, acute myelogenous leukemia; AUC, area under the curve; BMT, bone marrow transplant; CIS, clinically isolated syndrome; CNS, central nervous system; CSF, cerebrospinal fluid; DMT, disease modifying therapy; ECLIA, electrochemiluminescens immunosorbent assay; ELISA, enzyme linked immunosorbent assay; FC, fast converting; HAM, HTLV-I associated myelopathy; HC, healthy controls; IND, inflammatory neurological disease; ION, isolated opticus neuritis; LGI1, leucine-rich glioma-inactivated 1; LI, leptomeningeal involvement; LNB, Lyme Neuroborreliosis; Log2FC, Log2 fold change; MOGAD, myelin oligodendrocyte glycoprotein–associated disease; MS, Multiple Sclerosis; MSD, Meso Scale Discovery; n, number of participants per study group; NC, Neurologic controls; NIND, noninflammatory neurological disease; NHL, Non-Hodgkins lymphoma; NMDA, N-methyl-D-aspartate receptor; NMOSD, neuromyelitis optica spectrum disorder; NR, not reported; NPX, normalised protein expression; OBT, other brain tumors; OIND, other inflammatory neurological disease; PCNSL, primary central nervous system lymphoma; PIND, peripheral inflammatory neurological disease; PPMS, primary progressive multiple sclerosis; RRMS, relapsing-remitting multiple sclerosis; S, significant (p-value <0.05 or FDR <0.05); SAS, spinal anaesthesia subjects; SC, slow converting; sCD27, soluble CD27; TSP, tropical spastic paraparesis; y, years.*

Search strategy

PubMed (searched 7th of February 2025):

((cd27 OR soluble CD27 OR sCD27 OR TNFRSF7) OR (Proteomics AND (NMOSD OR NMO Spectrum Disorder OR neuromyelitis optica spectrum disorder OR MOGAD OR MOG Antibody Disease OR MOG antibody associated disease OR neuroborrel* OR CNS lymphoma OR central nervous system lymphoma OR neurosarcoidosis OR Central Nervous System Sarcoidosis OR MS OR multiple sclerosis OR RRMS OR relapsing remitting multiple sclerosis OR PPMS OR primary progressive multiple sclerosis OR meningitis OR encephalitis OR neurological immune-related adverse events OR (neurological AND checkpoint inhibitor)))) AND (cerebrospinal fluid OR CSF)

Results: 908 --> filter: no reviews, full text, english --> 783 results

Additional search for 2024/25 with no filter: 77 results

Embase (searched 7th of February 2025):

((cd27 OR soluble CD27 OR sCD27 OR TNFRSF7) OR (Proteomics AND (NMOSD OR NMO Spectrum Disorder OR neuromyelitis optica spectrum disorder OR MOGAD OR MOG Antibody Disease OR MOG antibody associated disease OR neuroborrel* OR CNS lymphoma OR central nervous system lymphoma OR neurosarcoidosis OR Central Nervous System Sarcoidosis OR MS OR multiple sclerosis OR RRMS OR relapsing remitting multiple sclerosis OR PPMS OR primary progressive multiple sclerosis OR meningitis OR encephalitis OR neurological immune-related adverse events OR (neurological AND checkpoint inhibitor)))) AND (cerebrospinal fluid OR CSF)

Results: 1213 → filter: removal of Medline records, full text, English, no reviews →141

Additional search for 2024/25 with only removal of Medline records: 28 results

Scopus (searched 7th of February 2025):

((cd27 OR soluble CD27 OR sCD27 OR TNFRSF7) OR (Proteomics AND (NMOSD OR NMO Spectrum Disorder OR neuromyelitis optica spectrum disorder OR MOGAD OR MOG Antibody Disease OR MOG antibody associated disease OR neuroborrel* OR CNS lymphoma OR central nervous system lymphoma OR neurosarcoidosis OR Central Nervous System Sarcoidosis OR MS OR multiple sclerosis OR RRMS OR relapsing remitting multiple sclerosis OR PPMS OR primary progressive multiple sclerosis OR meningitis OR encephalitis OR neurological immune-related adverse events OR (neurological AND checkpoint inhibitor)))) AND (cerebrospinal fluid OR CSF)

Results: 120 → filter: article (e.g. not reviews), English → 84 results

Additional search for 2024/25 with no filter: 14 results
